# Extensions to Extended Tight‐Binding Methods for Transition‐Metal Containing Systems

**DOI:** 10.1002/jcc.70346

**Published:** 2026-03-10

**Authors:** Siyavash Moradi, Rebecca Tomann, Martin Head‐Gordon, Christopher J. Stein

**Affiliations:** ^1^ TUM School of Natural Sciences and Catalysis Research Center, Department of Chemistry Technical University of Munich Garching Germany; ^2^ Pitzer Center for Theoretical Chemistry, Department of Chemistry University of California Berkeley California USA; ^3^ Atomistic Modeling Center, Munich Data Science Institute Technical University of Munich Garching Germany

## Abstract

Semi‐empirical quantum‐chemical methods such as extended tight‐binding (xTB) models are widely used for large‐scale simulations. Despite their popularity, their accuracy for transition‐metal containing systems is lower than, for example, closed‐shell organic molecules. In this work, we extend the Q‐Chem‐xTB framework with a geometric direct minimization (GDM) scheme for robust self‐consistent convergence and Hubbard correction (+U) to improve the description of local interactions and reduce self‐interaction errors similar to those characteristic of density‐functional theory calculations for transition‐metal complexes. The Hubbard correction term is integrated self‐consistently within the xTB Hamiltonian, allowing shell‐specific U values for each atom. The performance of Q‐Chem‐xTB+U is assessed for four benchmark sets of iron complexes, focusing on their spin‐state energetics. Sensitivity and optimization analyses of the spin parameters show that parameter tuning alone cannot systematically reduce the error or consistently recover correct spin ground‐state predictions across different datasets. In contrast, introducing the +U correction yields significant error reduction and improved electronic linearity with respect to fractional occupation, demonstrating that the correction fulfills its intended role of reducing self‐interaction error. However, the optimized U values remain system‐dependent, and the resulting improvements are only partially transferable. As a side effect, the +U correction stabilizes the self‐consistent field optimization by widening the HOMO–LUMO gap, thereby overcoming convergence instabilities of the conventional direct inversion of the iterative subspace (DIIS) scheme at low electronic temperatures.

## Graphical abstract

We present a new GFN2‐xTB implementation with a geometric direct minimization scheme and a Hubbard‐U correction. We demonstrate that the Hubbard correction improves linearity of the elctronic energy, stabilizes SCF convergence, and enables more accurate spin‐gap predictions in narrow application domains such as specific iron‐containing complexes.

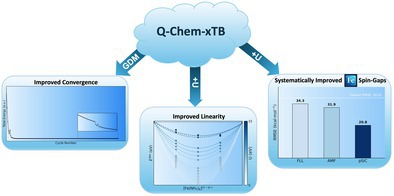

## 1 Introduction

Density functional tight‐binding (DFTB) [1] is a semi‐empirical quantum‐chemical method derived from density functional theory (DFT) [2, 3] that dramatically reduces the computational cost of simulations while preserving a quantum‐mechanical description of the (valence) electrons. A key milestone was the introduction of the self‐consistent‐charge DFTB (SCC‐DFTB) scheme by Elstner et al. in 1998, which incorporated charge self‐consistency to account for charge transfer and polarization effects [4]. Because of its efficiency—the method is capable of treating systems with hundreds or even thousands of atoms—DFTB became a popular tool for large‐scale simulations in materials science, biochemistry, and nanotechnology, offering orders‐of‐magnitude speed‐ups over full DFT calculations while maintaining reasonable accuracy for ground‐state properties [5, 6]. Over the years, DFTB has been continually improved and extended. Higher‐order corrections (such as DFTB3 variants) were introduced to include third‐order terms and better approximate the exchange‐correlation potential, reducing errors in bond lengths and energies for challenging cases [7, 8, 9]. Over time, extensive parameter sets have been systematically developed to cover wide regions of the periodic table and to enable applications across diverse classes of systems, including organic and biological molecules, transition‐metal complexes, and extended materials [10, 11, 12, 13, 14]. Nevertheless, the transferability of traditional DFTB parametrizations can be limited: the parameters are often fitted for specific bonding environments, resulting in reduced accuracy when these parameters are transferred to diverse chemical systems. This limitation motivated the development of extended tight‐binding (xTB) approaches that aim for more general applicability [15]. As another branch of the tight‐binding family, Grimme and co‐workers developed the xTB methods, designed to broaden applicability across a wide range of molecular systems [16, 17]. The first in this series, GFN1‐xTB (where “GFN” stands for Geometries, Frequencies, and Noncovalent interactions), retained the efficient DFTB‐like formalism but introduced a global parametrization strategy: the model parameters were optimized against reference data (geometries, vibrational frequencies, noncovalent interaction energies, etc.) for a wide range of molecular systems, rather than tuning to a narrow set of molecular species. This enormous optimization effort was partially enabled by adopting a framework in which most parameters were defined element‐wise rather than pairwise, thus reducing the overall number of parameters in the model. This yielded a semi‐empirical method that requires only minimal empiricism (no system‐specific reparameterization) yet achieves good accuracy across diverse applications in (mostly) organic chemistry. Building on this success, GFN2‐xTB, which was developed in 2019, introduced a more sophisticated treatment of electrostatics and additional element‐specific parameters, allowing it to handle not only main‐group elements but also many transition metals with improved accuracy. These GFN‐xTB methods exemplify the power of semi‐empirical approaches; by fitting to high‐quality ab initio reference data, they bridge the gap between fast but less accurate methods and the more reliable but expensive first‐principles calculations [18, 19]. The development of the xTB family has now culminated in the general‐purpose xTB method (g‐xTB) [20]. Conceived as a next‐generation semi‐empirical approach, g‐xTB is designed to achieve DFT‐like accuracy at tight‐binding speed, effectively combining the benefits of semi‐empirical quantum‐chemical methods and modern density functionals. While the above developments focused on improving the semi‐empirical electronic‐structure models, a parallel thread of research has addressed the deficiencies of the parent method, DFT, itself through the introduction of Hubbard U corrections. The DFT+U method was originally formulated in the early 1990s as an ad‐hoc correction to overcome the shortcomings of local‐density and gradient‐corrected DFT in describing Mott insulators and strongly correlated electrons [21, 22]. In essence, DFT+U adds an extra term to the DFT energy functional that penalizes fractional occupancy of specific orbitals (such as transition‐metal d or rare‐earth f orbitals), thereby reducing the self‐interaction error and enforcing a more localized electron density [23, 24]. This approach was refined by introducing rotationally invariant formalisms and combining the Hubbard U with density‐functional perturbation theory to determine optimal U values from first principles [25, 26, 27]. DFT+U has been shown to improve a wide array of predictions: it can stabilize the proper magnetic ordering in transition‐metal oxides [28, 29], correct charge ordering in mixed‐valence compounds [30], and generally improve the band gap and excitation energies for systems where localized electrons play a crucial role [25]. Analogous to DFT+U, a DFTB+U approach augments the SCC‐DFTB framework with a rotationally invariant on‐site correction that penalizes fractional occupations in localized orbitals, mitigating self‐interaction errors and over‐delocalization [31]. Within SCC‐DFTB, this is implemented in close analogy to LDA + U, such as the fully localized limit (FLL), around‐mean‐field (AMF) and pseudo‐self‐interaction correction (pSIC) formulation. Across molecules and materials, adding +U is often indispensable: for correlated oxides such as NiO, SCC‐DFTB+U opens a band gap and restores magnetic moments relative to plain SCC‐DFTB, while the derivative‐discontinuity correction further aligns the gap with experimental targets [31]. In f‐electron systems like ceria, a practical protocol linearly calibrates the position of the occupied Ce‐4f state versus U and then refits the repulsive potential to preserve structural energetics with respect to a chosen DFT+U reference [32] and in low‐dimensional materials, DFTB+U captures defect‐induced localization and magnetism [33]. These examples highlight how the Hubbard correction can recover missing localization effects and improve the description of strongly correlated subspaces across diverse materials classes. Building on this rationale, the same concept can be extended to transition‐metal complexes, where localized d electrons influence the delicate balance between competing spin configurations. The relative stability of spin states in transition‐metal complexes is a central property in chemistry, governing magnetic behavior, reactivity, and key thermodynamic processes such as spin crossover and catalysis. Because the spin‐state energy differences of transition‐metal containing systems are typically only a few kcal·mol^−1^, their accurate prediction remains one of the most demanding challenges for electronic‐structure methods [34]. DFT offers a practical balance between accuracy and computational cost, yet its performance depends sensitively on the choice of exchange–correlation functional and, in particular, the fraction of exact exchange included [35]. Hybrid functionals often overstabilize high‐spin states, whereas semilocal ones tend to favor low‐spin configurations due to over‐delocalization, making the correct energetic ordering difficult to achieve [36, 37]. To address these deficiencies, several approaches like the +U correction have been developed to reduce self‐interaction and delocalization errors, significantly improving the description of open‐shell transition‐metal complexes [37, 38, 39]. Although DFTB and xTB frameworks have been extended to handle open‐shell systems [40, 41, 42], studies explicitly addressing spin‐state energetics remain limited. A recent benchmark study [43] evaluated spin‐polarized GFN‐xTB methods for a representative set of 23 molecular and 20 periodic spin‐crossover systems. The analysis emphasized qualitative agreement with experimental and DFT ground states, employing the physically reasonable 0–10 kcal·mol^−1^ energy window. In this work, we extend the applicability of xTB methods to challenging transition‐metal systems by introducing the Hubbard correction and improved parameterization schemes within the Q‐Chem–xTB [42, 44] framework, which is based on the previously introduced, element‐specific parametrized GFN2‐xTB method. We first address the convergence difficulties commonly encountered for open‐shell transition‐metal complexes by integrating Q‐Chem's geometric direct minimization (GDM) scheme [45, 46] within the self‐consistent field procedure, ensuring robust convergence. Building upon this foundation, we perform a comprehensive benchmark of xTB variants across four different iron‐containing subsets of datasets—developed in the groups of Grimme [41] (also known as TM90S), Pantazis [47], Truhlar [48], and Ruiz [49]—to systematically evaluate their performance in predicting spin‐state energetics. Finally, we incorporate a Hubbard (+U) correction into the xTB Hamiltonian and optimize the corresponding U parameters. This additional on‐site term reduces self‐interaction and over‐delocalization errors in localized d‐orbitals, thereby improving both the energetic splittings and the electronic structure description of transition‐metal centers.

## 2 Theory

The total electronic energy in the Q‐Chem‐xTB implementation can be expressed as the sum of different contributions

(1) $$E_{\mathrm{tot}}=E_{\mathrm{rep}}+E_{\mathrm{EHT}}+E_{\mathrm{IES}}+E_{\mathrm{IXC}}+E_{\mathrm{AES}}+E_{\mathrm{AXC}}+E_{\text{spin}}+E_{\text{Fermi}}.$$

$E_{\mathrm{rep}}$ represents the repulsion energy, $E_{\mathrm{EHT}}$ is the extended Hückel‐type term describing covalent interactions, $E_{\mathrm{IES}}$ and $E_{\mathrm{IXC}}$ correspond to the isotropic electrostatic and exchange–correlation contributions, respectively, while $E_{\mathrm{AES}}$ and $E_{\mathrm{AXC}}$ denote the anisotropic corrections that capture the directionality of multipole interactions. $E_{\text{spin}}$ accounts for the spin‐polarization energy, and $E_{\text{Fermi}}$ refers to the finite‐temperature entropic correction due to Fermi smearing [50]. Note that an unrestricted formalism was used throughout this work. All terms and the corresponding contributions to the Fock matrix have been discussed in the original GFN2‐xTB article such that we highlight only new terms and those where we attempt a parameter optimization. The spin‐polarization energy belongs to the latter class and can be written as

(2) $$E_{\text{spin}}=\frac{1}{2}\sum_{A}^{N}\sum_{l\in A}\sum_{l^{\prime }\in A}p_{\mathrm{Al}}p_{Al^{\prime }}W_{\mathrm{Al}l^{\prime }},$$

where $p_{\mathrm{Al}}$ denotes the difference between the α‐ and β‐spin Mulliken populations for the atomic shell l on atom A and $W_{\mathrm{Al}l^{\prime }}$ is the spin parameter. As an additional contribution, we further incorporate an on‐site Hubbard correction to account for the self‐interaction and over‐delocalization. In analogy to previously established formulations, three choices are implemented: the FLL [51], the around mean field (AMF) [52], and the pseudo self‐interaction correction (pSIC) [53].

Their corresponding energy expressions are given by

(3) $$E_{U}^{\mathrm{FLL}}=-\alpha \sum_{A}^{N}\sum_{l\in A}\frac{U_{l}}{2}\sum_{\sigma }\sum_{\mu \nu \in l}\left[{\left(n_{\mu \nu }^{\sigma }\right)}^{2}-n_{\mu \nu }^{\sigma }\right],$$

(4) $$E_{U}^{\text{pSIC}}=-\alpha \sum_{A}^{N}\sum_{l\in A}\frac{U_{l}}{2}\sum_{\sigma }\sum_{\mu \nu \in l}{\left(n_{\mu \nu }^{\sigma }\right)}^{2},$$

(5) $$E_{U}^{\mathrm{AMF}}=-\alpha \sum_{A}^{N}\sum_{l\in A}\frac{U_{l}}{2}\sum_{\sigma }\sum_{\mu \nu \in l}{\left(\delta n_{\mu \nu }^{\sigma }\right)}^{2},$$

where $U_{l}$ is the Hubbard parameter for the atomic shell l, $\sigma \in \left\{↑,↓\right\}$ labels the two spin channels and α is an empirical scaling factor, typically set to 0.5 [54]. The corresponding Fock contributions take the following form [31]

(6) $$F_{\mu \nu }^{\sigma ,\mathrm{FLL}}=-\alpha U_{l}\left(n_{\mu \nu }^{\sigma }-\frac{1}{2}\delta_{\mu \nu }\right),$$

(7) $$F_{\mu \nu }^{\sigma ,\text{pSIC}}=-\alpha \frac{U_{l}}{2}\;n_{\mu \nu }^{\sigma },$$

(8) $$F_{\mu \nu }^{\sigma ,\mathrm{AMF}}=-\alpha U_{l}\;\delta n_{\mu \nu }^{\sigma },$$

where δn is

(9) $$\delta n_{\mathrm{\mu v}}^{\sigma }=n_{\mathrm{\mu v}}^{\sigma }-\frac{\sum_{v}n_{\mathrm{vv}}^{\sigma }}{2l+1}\delta_{\mathrm{\mu v}}.$$

The $n_{\mu \nu }^{\sigma }$ denotes the occupation matrix which is constructed from the density matrix $P_{\mu \nu }^{\sigma }$ and the overlap matrix $S_{\mu \nu }$ via

(10) $$n_{\mu \in A,\nu \in A}^{\sigma }=\frac{1}{2}\sum_{B}\sum_{\tau \in B}\left(S_{\mu \tau }\;P_{\tau \nu }^{\sigma }+P_{\mu \tau }^{\sigma }\;S_{\tau \nu }\right).$$

To achieve improved spin‐state energetics, both the spin‐polarization parameters $W_{\mathrm{Al}l^{\prime }}$ and the on‐site Coulomb parameters ($U_{l}$) are systematically optimized. As a first step, a sensitivity analysis based on the Sobol variance decomposition method [55] is performed using the SALib library [56] to quantify the relative influence of each parameter on the computed spin‐state energy gaps. This analysis enables the identification of the most impactful parameters, allowing the optimization to focus on a reduced and physically meaningful subspace of the parameter set [42, 57]. Subsequently, a two‐stage optimization strategy is employed; an initial coarse search is conducted using the derivative‐free Powell algorithm to efficiently explore the parameter landscape without relying on gradient information. The resulting parameters serve as a starting point for a refined optimization using L‐BFGS‐B, which leverages gradient information to ensure stable optimization.

## 3 Results and Discussion

To improve the spin‐gap prediction by optimizing the spin‐splitting and Hubbard parameters, convergence of the electronic‐structure calculation must be guaranteed for a range of systems and choices of parameters. Since for low‐gap systems, standard convergence accelerating protocols such as the direct inversion of the iterative subspace (DIIS) [58, 59] do not guarantee convergence, improved algorithms are required.

### 3.1 SCF Convergence

Self‐consistent‐field (SCF) convergence in xTB methods can be fragile, particularly for (transition‐)metal systems with near‐degenerate orbitals. DIIS and Broyden [60] methods generally perform well for main‐group or organic molecules but are sometimes unstable due to large charge oscillations in systems with small HOMO–LUMO gaps or near‐degenerate states, features that are common in transition‐metal clusters and open‐shell complexes [61]. Such cases frequently lead to oscillatory or divergent SCF behavior, even when electronic smearing is applied through Fermi smearing at 300 K. To address these issues, we have coupled the GDM approach [45, 46] with Q‐Chem‐xTB. Unlike DIIS, which uses an extrapolation scheme, GDM minimizes the electronic energy directly on the manifold of orthonormal orbitals (Grassmann manifold) by following the energy gradient. It adaptively adjusts the step size to ensure stable descent and refines the molecular orbitals until a true minimum is reached. This makes GDM inherently more stable—and often slower—than DIIS but shows reliable convergence even for challenging transition‐metal systems. Table 1 compares DIIS and GDM convergence in Q‐Chem‐xTB against the Broyden method that is implemented in the original GFN2‐xTB implementation for a LiF molecule over a range of bond lengths. Since we do not include the D4 correction in Q‐Chem‐xTB we manually removed the D4 dispersion contribution from the GFN2‐xTB source code, both from the total energy and Fock‐matrix contributions, to enable a one‐to‐one comparison with Q‐Chem‐xTB. For converging situations, we see identical results within the convergence threshold. For challenging bond‐stretched scenarios, GDM requires significantly fewer iterations and converges reliably even for the almost dissociated molecule at 4 Å, where both DIIS and Broyden convergence acceleration fail.

**TABLE 1:** Comparison of SCF convergence for LiF at various bond lengths using different optimization methods.

Bond length (Å)	Energy (Hartree)			Number of iterations		
	Q‐Chem	GFN2 (D4 off)	GFN2	Q‐Chem DIIS	Q‐Chem GDM	GFN2 Broyden
1.25	−5.04598	−5.04598	−5.04616	5	5	9
1.40	−5.07535	−5.07535	−5.07552	5	5	9
1.60	−5.07885	−5.07885	−5.07902	5	7	9
2.00	−5.05040	−5.05040	−5.05058	6	7	12
2.50	−5.01033	−5.01033	−5.01082	8	9	24
3.00	−4.97685	−4.97685	−4.97735	14	9	39
4.00	−4.93505	—	—	Failed	25	Failed

*Note:* Energies and iteration counts are shown for Q‐Chem‐xTB (with DIIS and GDM), GFN2‐xTB with and without D4 dispersion. All GFN‐xTB calculations in this paper are performed using version 6.7.1 of the xTB program. Both xTB and Q‐Chem use the same convergence threshold ($10^{-6}$), maximum iteration limit (250) and electronic temperature (300 K).

**FIGURE 1:**
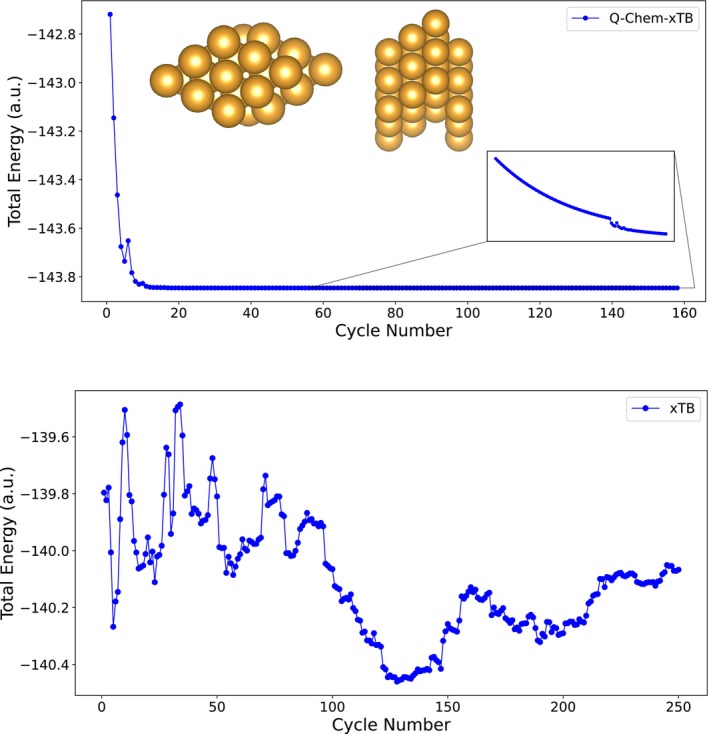
Convergence for a Au_36_‐cluster with xTB and GDM in Q‐Chem‐xTB.

In Figure 1 we compare the convergence behavior of GDM in Q‐Chem and the Broyden method in xtb for an even more challenging Au cluster (Au_36_). GDM in Q‐Chem‐xTB converges smoothly, eventually reaching a stable minimum without significant oscillations. Although the convergence is slow, we observe a mostly smooth energy decrease until the convergence threshold is reached. In contrast, the corresponding xtb calculation using Broyden mixing features strong oscillations at considerably higher energies and no convergence could be reached. It is also worth noting that the GDM optimization shown here was performed at zero electronic temperature to provide the most challenging scenario, whereas we applied an electronic temperature of 300 K in the xtb calculation. The corresponding GDM optimization at 300 K converges in only 36 iterations to the same threshold (see SI).

### 3.2 Benchmark of Fe Spin‐State Gaps for Extended Tight‐Binding Variants

Iron complexes are highly relevant due to the abundance of the metal and at the same time often challenging due to many accessible spin‐states. Therefore, we deem a set of iron‐containing transition‐metal complexes a suitable challenge for spin‐gap predictions with xTB methods. We hence compiled a dataset containing 53 spin gaps of iron complexes from previous studies published by the labs of Grimme [41], Ruiz [49], Pantazis [47], and Truhlar [48]. We will refer to these subsets of the total dataset using the names of the main PI in the following. We first tested the performance of various xTB methods on this dataset. For all four datasets, we used the molecular structures provided in the original publications, without further re‐optimization. Concerning the reference method, we follow the recommendations of the original publications and further provide a non‐xTB result as a baseline for the accuracy that can be expected. In the case of Ruiz and Truhlar, the chosen reference methods were selected because they reproduce the correct experimental ground state in all cases. The significant deviation of the non‐xTB baseline results from the reference calculations highlights the difficulty of spin‐gap prediction, even for advanced electronic‐structure methods. Even for optimal parameters, it is not to be expected that the xTB methods perform better than DFT methods. As an additional note, the width of the spin gaps deviates strongly between the datasets, such that the absolute errors should not be compared directly. The results in Table 2 provide a comprehensive overview of how different tight‐binding methods perform on the different datasets. For all datasets, the spin‐polarized spGFN2‐xTB variant improves over the less complex spGFN1‐xTB method. This is not directly obvious for the Truhlar dataset but the RMSE approaches that of the DFT baseline, which indicates an improvement toward the method the xTB tries to approximate. Overall, spGFN2‐xTB also predicts more ground states correctly than spGFN1‐xTB. The current Q‐Chem‐xTB implementation does not include any dispersion interactions, explaining the difference between the results obtained with this method and the spGFN2‐xTB data. Reducing the electronic temperature to 0 K—a situation where convergence is now possible due to the GDM optimization—leads to negligible changes.

**TABLE 2:** Performance of xTB methods on spin‐state energy gaps across four benchmark datasets.

Method	Grimme	Ruiz	Pantazis	Truhlar
spGFN1‐xTB	48.61 (8/11)	8.08 (5/8)	13.16 (10/12)	20.96 (16/22)
spGFN2‐xTB	43.71 (9/11)	6.24 (6/8)	9.55 (12/12)	24.75 (15/22)
Q‐Chem‐xTB	38.01 (9/11)	4.60 (7/8)	10.78 (11/12)	25.36 (16/22)
Q‐Chem‐xTB (T=0)	37.65 (9/11)	4.18 (7/8)	10.76 (11/12)	25.08 (16/22)
Q‐Chem‐xTB‐D3	38.33 (10/11)	3.73 (8/8)	11.75 (11/12)	26.25 (16/22)
g‐xTB	36.58 (10/11)	5.64 (8/8)	20.87 (10/12)	23.16 (15/22)
Non‐xTB baseline	13.32 (9/11) PBE‐D4	2.68 (8/8) TPSSh‐QZVP	7.75 (8/12) DLPNO‐CCSD	24.06 (17/22) PBE‐TZVP
Range (kcal·mol^−1^)	$\left[-29.4,47.5\right]$	$\left[5.3,11.4\right]$	$\left[-14.1,29.6\right]$	$\left[-29.0,41.5\right]$
Reference	TPSSh‐D4/QZVPP	TPSSh/TZVP	CASPT2/CC	PW6B95/TZVP

*Note:* RMSE in kcal·mol^−1^; gap sign predictions: Correct/total in parentheses. The non‐xTB values are taken from the original papers.

For a pragmatic inclusion of dispersion interactions into our implementation, we added the density‐independent D3 dispersion correction in Q‐Chem‐xTB‐D3 with Becke–Johnson damping and PBE parameters [62, 63]. The discrepancy between the D3 and D4 correction is still sizable but is out of scope for a discussion in this article. As a further point of comparison, we included the recently published model g‐xTB [20] in this benchmark. Somewhat surprisingly, the results obtained with this new variant were slightly to significantly (Pantazis dataset) worse than those obtained with the other methods.

### 3.3 Spin Parameter Optimization

**FIGURE 2:**
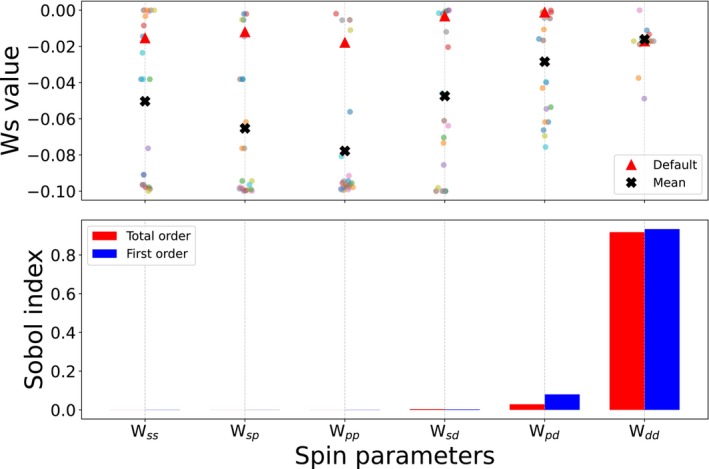
Sensitivity analysis and individual parameter optimization for Truhlar dataset.

**FIGURE 3:**
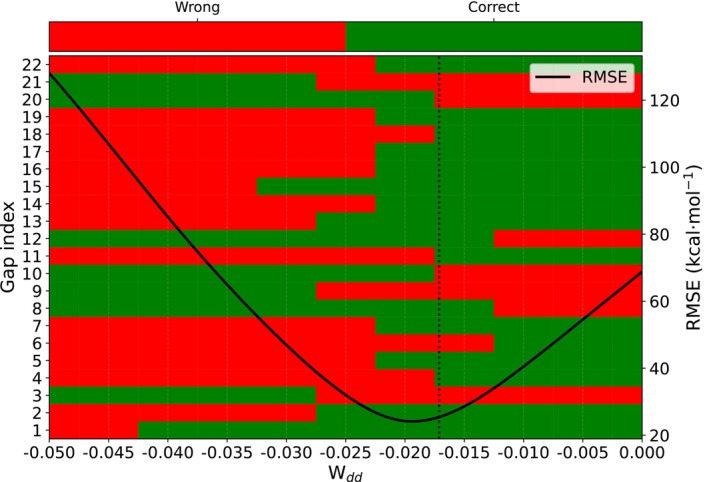
Heatmap illustrating the dependence of spin‐gap sign prediction on the $W_{\mathrm{dd}}$ spin parameter. Each horizontal row corresponds to a single spin gap from the Truhlar dataset (*y*‐axis), evaluated across the range of $W_{\mathrm{dd}}$ values (*x*‐axis). The color of each cell indicates the correctness of the sign prediction: Green denotes a correct sign (matching the reference), and red denotes an incorrect prediction. The vertical dotted line marks the default $W_{\mathrm{dd}}$ value. The line shows the RMSE for each $W_{\mathrm{dd}}$ value.

With these initial results, we investigated whether optimization of the spin‐polarization parameters, as described in the theory section, allowed us to improve the predictions. Table 3 summarizes the results for an optimization on each individual dataset. The improvement was only marginal despite the fact that for each given spin gap, an individual optimization allowed us to remove any error with respect to the reference results. To understand this limited gain, we performed a Sobol sensitivity analysis of the spin parameters (see Figure 2, lower panel for the Truhlar dataset), which revealed that, as expected, only the parameter for the spins in d‐orbitals, $W_{\mathrm{dd}}$, influences the prediction significantly. In the upper panel of Figure 2, we plot the optimized spin‐polarization parameters for each individual spin gap. While we see a significant variance, the Sobol analysis shows us that for most parameters even varying the value used in a given calculation will not significantly alter the spin‐gap prediction. For the relevant $W_{\mathrm{dd}}$, however, we find that the mean of the optimized values almost perfectly coincides with the default parameter, explaining the little gain in accuracy that can be achieved by this optimization. A similar trend was also observed for the Grimme dataset (see Supporting Information). This highlights the limited transferability that can be achieved by parameter tuning for these systems. To further probe this behavior, we systematically varied $W_{\mathrm{dd}}$ within the range $\left[-0.05,0.0\right]$ and monitored the number of correctly predicted spin‐gap signs for the Truhlar dataset (Figure 3). This analysis further confirms that while local tuning of $W_{\mathrm{dd}}$ can improve individual cases, a globally transferable set of spin parameters cannot be achieved.

**TABLE 3:** RMSE values for different datasets with default and optimized spin parameters.

	RMSE (Grimme)	RMSE (Ruiz)	RMSE (Pantazis)	RMSE (Truhlar)
Default	38.01 (9/11)	4.60 (7/8)	10.78 (11/12)	25.36 (16/22)
Optimized	37.67 (9/11)	3.65 (7/8)	10.39 (11/12)	24.56 (16/22)

### 3.4 Q‐Chem‐xTB With Hubbard U Correction

The persistently inaccurate transition‐metal spin‐gap prediction, particularly for iron complexes, can be partially attributed to self‐interaction errors (SIE) that lead to an over‐delocalization of d electrons. To mitigate this deficiency, we extended the Q‐Chem‐xTB framework with a Hubbard on‐site correction, following three common formulations, FLL, AMF, and pSIC, as detailed in the theory section. We have optimized the $U_{s}$, $U_{p}$, and $U_{d}$ parameters for iron and for each variant using the Grimme dataset, and the resulting values and corresponding root mean square errors (RMSEs) are summarized in Table 4. A Sobol sensitivity analysis (see SI) revealed that $U_{d}$ dominates the variance in spin‐gap predictions as expected, whereas $U_{s}$ has no influence. As seen in the table, all three variants lead to lower RMSE values compared to the uncorrected model, indicating that introducing on‐site corrections systematically improves the description of spin‐state energetics. Notably, only the pSIC scheme not only reduces the overall RMSE most significantly (from 38.01 to 20.76 kcal·mol^−1^) but also increases the number of correctly predicted spin ground states, demonstrating a qualitative improvement in spin‐state ordering. This enhanced performance aligns with previous observations that the pSIC formulation provides superior results compared to FLL in DFTB+U studies, such as for carbon adatoms [64]. The findings of Sanna et al. further support the superior performance of pSIC [65]. In their DFTB study of ErN, pSIC was the only correction that improved the bulk modulus without worsening the lattice constant, yielding a more balanced description of structural properties. They also showed that pSIC correctly shifts the occupied 4f states downward, consistent with the behavior expected for correlated materials, while avoiding the excessive distortion of unoccupied states observed in the FLL variant.

**TABLE 4:** Optimized Hubbard U parameters and resulting RMSE values for the Grimme dataset. The RMSE in the absence of the Hubbard correction is 38.01 kcal·mol^−1^.

	Optimized $U_{s}$ (Ha)	Optimized $U_{p}$ (Ha)	Optimized $U_{d}$ (Ha)	RMSE (kcal·mol^−1^)
FLL	0.3543	0.0000	0.07549	34.32 (9/11)
AMF	0.2998	0.23783	0.07767	31.94 (9/11)
pSIC	0.3674	0.14036	0.14627	20.76 (10/11)

To test whether the U values optimized on the Grimme set yield transferable improvements beyond their training domain, we applied the pSIC‐optimized $\left(U_{s},U_{p},U_{d}\right)$ to the other three datasets. The cross‐set performance (Table 5) shows very poor transferability: RMSE values increase markedly on all three datasets and the number of correctly predicted spin‐gap signs drops. This demonstrates that the U parameter tuned on the Grimme dataset is able to adjust the spin‐gap prediction but is extremely system dependent, such that the accuracy decreases when the optimized parameters are used for other datasets, where different ligands and oxidation states dominate.

**TABLE 5:** Comparison of RMSE values (in kcal·mol^−1^) for different datasets with and without the Hubbard +U correction with the U parameters optimized on the Grimme dataset.

	RMSE (Ruiz)	RMSE (Pantazis)	RMSE (Truhlar)
No +U	4.60 (7/8)	10.78 (11/12)	25.36 (16/22)
With +U	43.21 (1/8)	18.97 (6/12)	43.33 (5/22)

**FIGURE 4:**
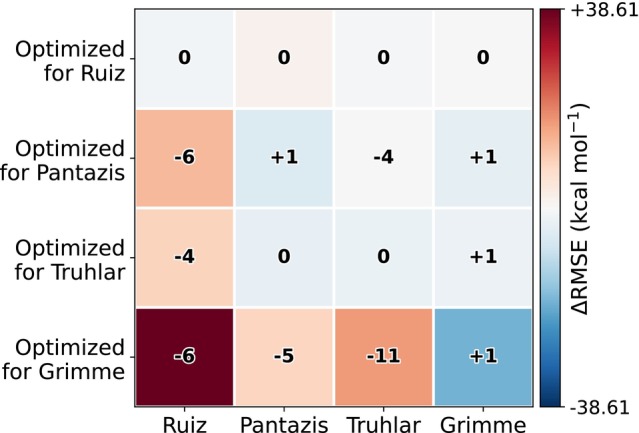
Cross‐dataset transferability of optimized $U_{d}$ and $W_{\mathrm{dd}}$ parameters. Blue regions indicate improved accuracy relative to the default RMSE (ΔRMSE), while red regions denote less accurate predictions. Values in the middle of each square show the change in correctly predicted spin‐gap signs for each case relative to the number obtained with the default parameters.

Motivated by the results of the Sobol analysis, from which we identified $W_{\mathrm{dd}}$ and $U_{d}$ as the most influential parameters, we performed two‐parameter optimizations on all datasets and then assessed the respective cross‐transferabilities (Figure 4). In every case, the RMSEs on the dataset for which these parameters were optimized decrease (diagonal elements), confirming that one can tune the parameters to fit the specific dataset. However, two observations emerge: The correct spin‐ground state prediction is almost not improved. In only two cases a single additional correct ground state is predicted for another dataset; the other two sets show no changes in sign counts despite lower RMSE. That the worst results are obtained for transferability to the Ruiz dataset is not surprising since this dataset featured much smaller spin gaps than the other three and should hence be much more sensitive. Certainly, for an optimization with an objective function that aims to improve the number of correct ground‐state predictions this could be improved, but when aiming to lower the total spin‐gap RMSE for a dataset, we do not see significant optimization. As a second observation, we note that transferability remains poor in all cases. Optima obtained on one dataset do not carry over to the others; applying them degrades either RMSE, sign counts, or both. Apparently, while local minima can be found for chemically similar systems, a global optimum is difficult to achieve or is already close to the default values. These findings are consistent with prior ab initio studies on Hubbard corrections that say the Hubbard U parameter is intrinsically non‐transferable, as its value depends sensitively on the local chemical environment, oxidation state, and even the basis set used to define the correlated subspace [66, 67]. While the good performance of the default parameters is not surprising for the carefully optimized parameters of the initial GFN2‐xTB implementation, the parameters optimized here are either absent in the original formulation (Hubbard U parameters), or derived from the parent DFT method using the PBE functional (spin‐polarization parameters). We would have assumed that a careful, element‐specific reparameterization of these parameters within the full framework would yield global improvement, but we find that this is not the case, at least for this specific dataset of iron compounds.

**FIGURE 5:**
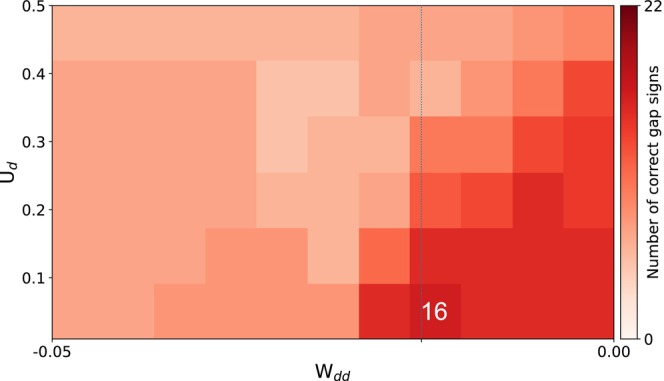
Spin‐gap sign accuracy as a function of $W_{\mathrm{dd}}$ and $U_{d}$ (both in Hartree) for the Truhlar dataset.

As an additional check in Figure 5, we varied $W_{\mathrm{dd}}$ and $U_{d}$ over broad ranges and inspected for which combination of parameters we obtain the largest number of correct spin‐gap signs. The best combination of parameters yields 16 correct predictions out of 22 and coincides with the default parameters.

To check if a first‐principles approach for the determination of the Hubbard parameter yields better results than the empirical optimization or fitting procedure, we determined the U parameters based on the curvature of the energy with respect to fractional occupation [68, 69, 70], as previously applied in molecular DFT+U by Kulik and co‐workers [71]. In this approach, the effective Hubbard U is estimated from the deviation from piecewise linearity of the total energy, expressed as the difference between the HOMO eigenvalue of the $\left(N+1\right)$‐electron system and the LUMO eigenvalue of the N‐electron system ($\varepsilon_{N+1}^{\text{HOMO}}-\varepsilon_{N}^{\text{LUMO}}$). We implemented this self‐consistent scheme within the Q‐Chem‐xTB+U framework and used it for the Grimme dataset. The resulting U values were consistently larger than those obtained from the empirical optimization. Consequently, the spin‐gap RMSE increased significantly, from 38.01 to about 60 kcal·mol^−1^, in contrast to the reduction to ∼ 20 kcal·mol^−1^ achieved with the empirically optimized U. As previously observed in DFT+U studies for spin‐state energies, this behavior is not unique to Q‐Chem‐xTB+U. In DFT+U, self‐consistent U values were also found to be systematically overestimated, typically 1–2 eV higher than the empirically optimal ones, leading to poor agreement for low‐spin configurations [37]. Similarly, in the study of biased spin‐state energetics of Fe(II) complexes [39], it was shown that self‐consistent U schemes tend to overstabilize high‐spin states because both the Hubbard energy term and the computed U are larger for LS states, resulting in their over‐penalization.

### 3.5 Self Interaction Error

**FIGURE 6:**
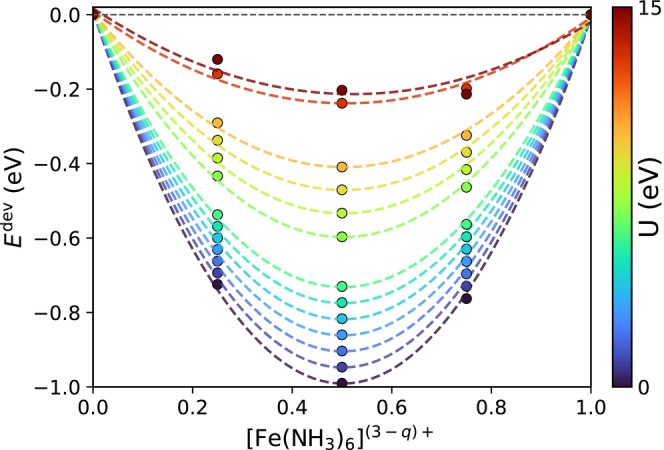
Deviations from linearity ($E^{\mathrm{dev}}$) as the charge is varied from +3 to +2 (i.e., q from 0 to 1) for the high‐spin [Fe(NH_3_)_6_]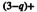 complex (sextet → quintet). Filled circles represent total energies from fractional‐charge calculations, while dashed lines show cubic spline interpolations based on integer‐charge frontier orbital energies. The color bar indicates the applied Hubbard U values for each curve. The horizontal gray dashed line marks zero deviation from linearity. The deviation is defined as $E^{\mathrm{dev}}\left(q\right)=E\left(q\right)-\Delta E\;q,$ where ΔE is the total energy difference between the q=1 (quintet) and q=0 (sextet) state.

SIE is a well‐known limitation of DFT and xTB methods, particularly for open‐shell transition‐metal systems, where the over‐delocalization of d electrons can lead to artificially small HOMO–LUMO gaps and unstable SCF behavior. In spin‐polarized GFN2‐xTB [41], it has been noted that disabling the electronic temperature (i.e., Fermi smearing) leads to severe convergence failures due to these narrow gaps. This problem was also identified during the development of g‐xTB [20], where the small gap was attributed to SIE and over‐delocalization, and a non‐local Fock exchange correction was introduced to widen the gap and allow SCF convergence at zero temperature with a DIIS implementation. The Hubbard‐U correction has a similar effect in our Q‐Chem‐xTB implementation: with increasing U parameter, the HOMO–LUMO gap increases systematically, indicating a reduction of SIE and a stabilization of localized orbitals. As a result, SCF iterations using DIIS that previously failed to converge at zero temperature now converge reliably. Table 1 in the SI summarizes the fraction of DIIS convergence failures across all the datasets at zero temperature, both with and without the U correction. Without U, convergence failures occur frequently, up to 51% for the Truhlar set and 41% for the Grimme set, whereas with the +U term, all systems converge robustly. The corresponding increase in HOMO–LUMO gaps is also shown in the SI. We also analyzed how the Hubbard correction influences the linearity of the total energy with respect to fractional electron occupation, a direct measure of the self‐interaction error [26, 72, 73]. In the exact theory, the total energy varies linearly between integer occupations, whereas approximate functionals typically show a convex curvature that reflects electron over‐delocalization [69, 74]. It has been demonstrated that DFT+U can partially restore this linear behavior, though standard atomic‐orbital projectors tend to under‐correct in systems with strong metal–ligand hybridization, where molecular‐orbital‐based projectors perform better [71]. In our calculations on the[Fe(NH_3_)_6_]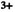 complex (see Figure 6), the total‐energy curve at U=0 displays a strong convex deviation of about −1 eV at half‐electron removal. Increasing U progressively flattens the profile. This improvement confirms that, also with the xTB framework, the Hubbard term effectively suppresses delocalization and enforces improved linearity. Nevertheless, the small residual curvature at large U values suggests incomplete localization of the fractional charge, consistent with the interpretation that orbital mixing between Fe 3*d* and ligand N 2*p* states limits the performance of the corrections. Although full piecewise linearity cannot be completely achieved within this framework, the application of the Hubbard term substantially reduces the curvature and brings the energy–charge relationship much closer to the ideal linear behavior, thereby representing a significant improvement over the uncorrected case.

## 4 Conclusion

In conclusion, we present a systematic extension of the Q‐Chem‐xTB framework by integrating the GDM algorithm for improved SCF convergence and the Hubbard correction +U for improving the treatment of localized electronic subspaces. Both developments address long‐standing challenges in semi‐empirical quantum methods when applied to transition‐metal systems, where narrow HOMO–LUMO gaps, strong self‐interaction errors, and near‐degenerate states often lead to numerical instabilities and inaccurate spin‐state energetics. The implementation of GDM significantly improves SCF convergence behavior compared to conventional DIIS in Q‐Chem‐xTB or Broyden mixing in GFN2‐xTB. For metal clusters and open‐shell complexes, GDM eliminates oscillatory behavior and achieves smooth energy descent, providing a stable foundation for subsequent energy and property calculations. After establishing robust convergence, we benchmarked various xTB‐based methods for iron spin‐state energetics using four standard datasets (Grimme, Ruiz, Pantazis, and Truhlar). The results show that while spin‐polarized GFN2‐xTB and Q‐Chem‐xTB achieve reasonable accuracy, parameter optimization for the spin‐polarization term alone cannot substantially reduce errors or systematically recover correct spin‐gap signs across datasets. To address the self‐interaction error and over‐delocalization of d electrons, we introduced a Hubbard +U correction into the xTB Hamiltonian. The correction was implemented following three common formulations (FLL, AMF, and pSIC), and the corresponding U parameters for the s, p, and d shells were optimized. Among these, the pSIC form yielded the most significant improvement. Unfortunately but in line with previous research on DFT+U, when these optimized U values were transferred to other datasets, their performance deteriorated, indicating that the optimal correction depends strongly on the local chemical environment. In addition to improving accuracy, the +U correction also enhanced SCF stability by widening the HOMO–LUMO gap. Furthermore, we demonstrated that increasing U progressively restores the linearity of the total energy with respect to a fractional change in total electron number, confirming the physical consistency of the correction in reducing the self‐interaction error.

In future work, we will investigate how both the GDM implementation and the Hubbard+U correction improve (periodic) calculations on metals and metallic nanoclusters.

## Conflicts of Interest

The authors declare the following competing financial interest(s): M.H.‐G. is a part‐owner of Q‐Chem Inc., whose software was used for all developments and calculations reported here.

## Supporting information


**Data S1:** jcc70346‐sup‐0001‐Supinfo.pdf.

## Data Availability

All benchmark datasets used in this study are publicly available and cited in the manuscript.
