# Supplementary material for: Extensions to Extended Tight‐Binding Methods for Transition‐Metal Containing Systems
Source: J Comput Chem. 2026 Mar 10;47(7):e70346. doi: 10.1002/jcc.70346 (PMC12973268; doi:10.1002/jcc.70346)
Supplement: Supplementary file 1 — Data S1: jcc70346‐sup‐0001‐Supinfo.pdf. [file JCC-47-0-s001.pdf]

Supporting information: Extensions to extended tight-binding methods for transition-metal containing systems

Siyavash Moradi,[†] Rebecca Tomann,[‡] Martin Head-Gordon,[‡] and Christopher J. Stein*,[†,¶]

†*Technical University of Munich, TUM School of Natural Sciences and Catalysis Research Center, Department of Chemistry, Lichtenbergstr. 4, 85748 Garching, Germany*

‡*Pitzer Center for Theoretical Chemistry, Department of Chemistry, University of California, Berkeley CA 94720, USA*

¶*Atomistic Modeling Center, Munich Data Science Institute, Technical University of Munich, Walther-von-Dyck Str. 10, 85748 Garching, Germany*

E-mail: christopher.stein@tum.de

1. Convergence comparison between DIIS at 0 and 300 K and GDM at 300 K for Au_{36}

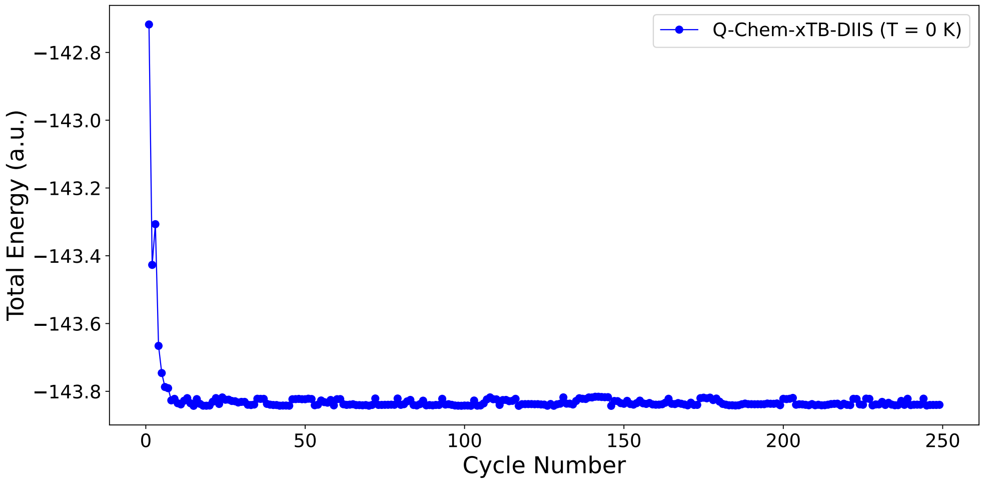

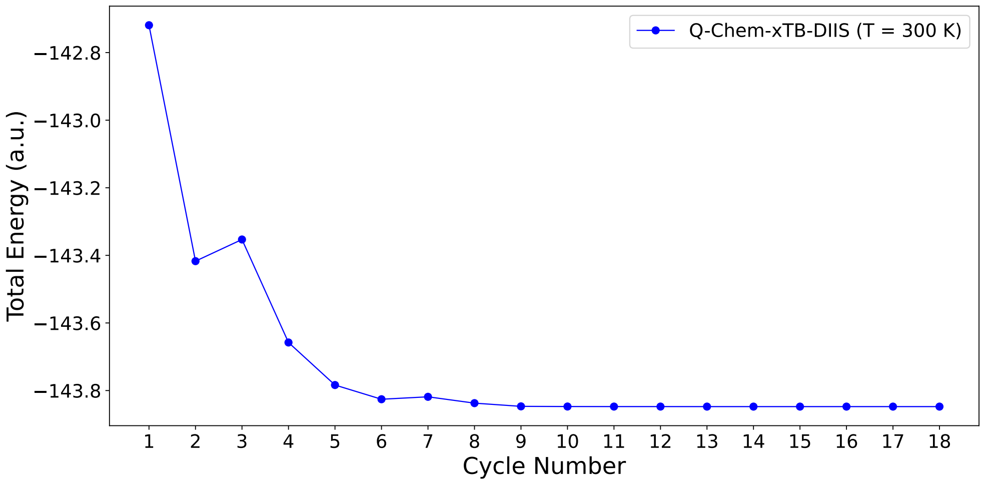

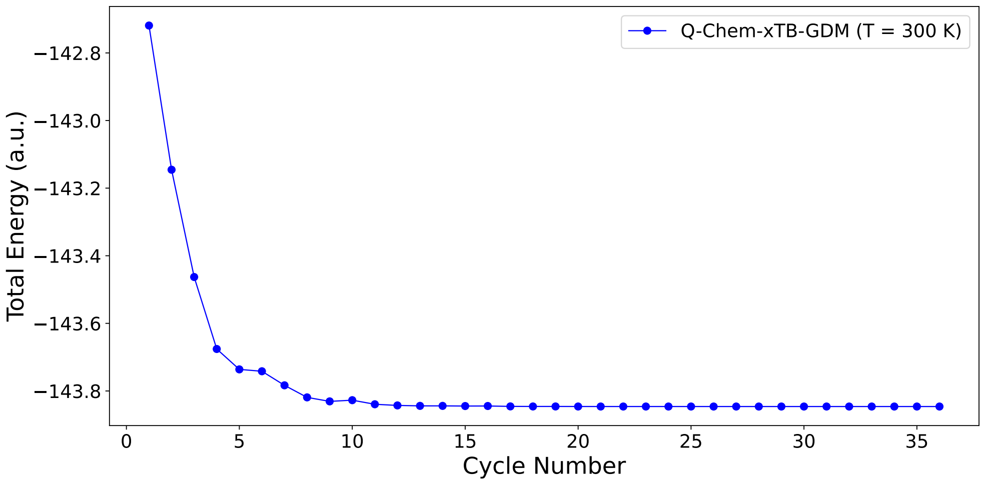

2. Individual spin gap optimization for Grimme dataset

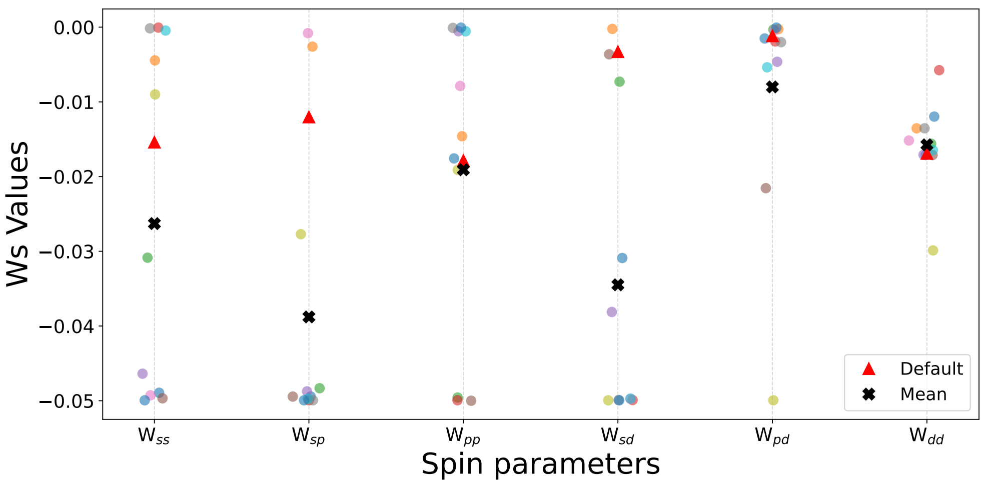

3. Sensitivity analysis for Hubbard parameters using Grimme dataset

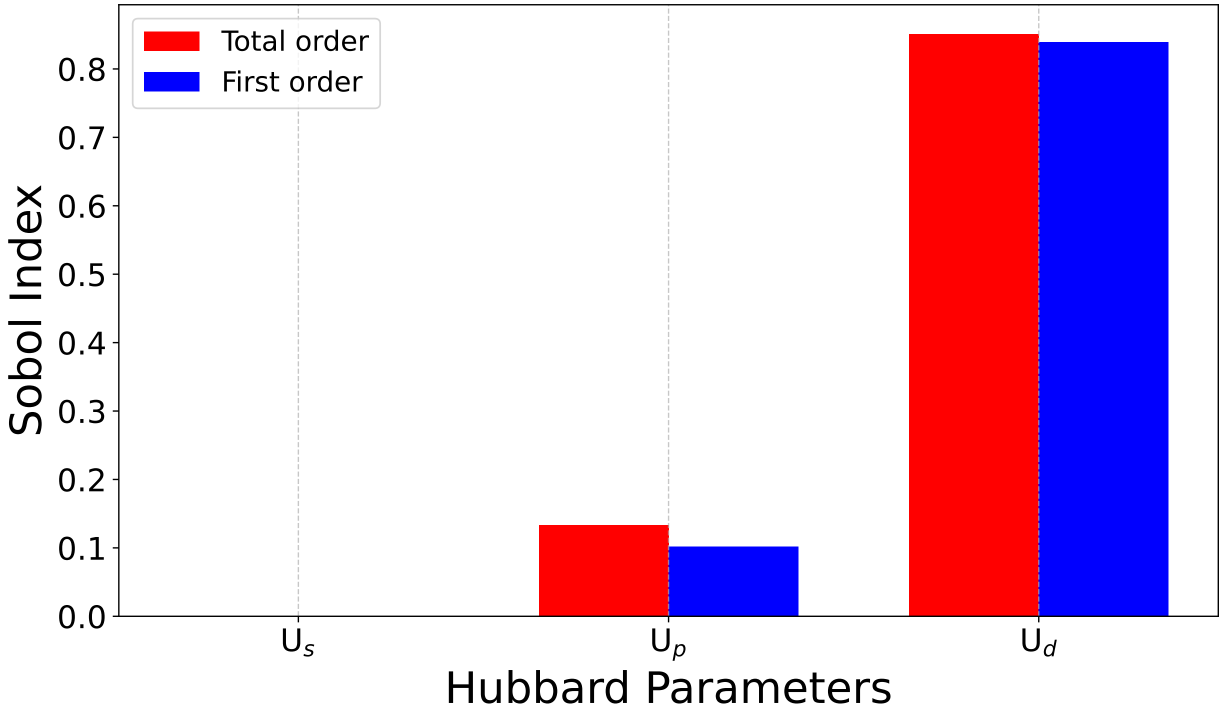

4. DIIS convergence failure rates with and without the Hubbard $+U$ correction at zero temperature

	DIIS Failure (%) Without $+U$	DIIS Failure (%) With $+U$
Truhlar	51.0	0.0
Grimme	41.0	0.0
Ruiz	6.0	0.0
Pantazis	0.0	0.0

5. HOMO-LUMO gap for each iron structure in the Grimme dataset with and without $+U$ correction

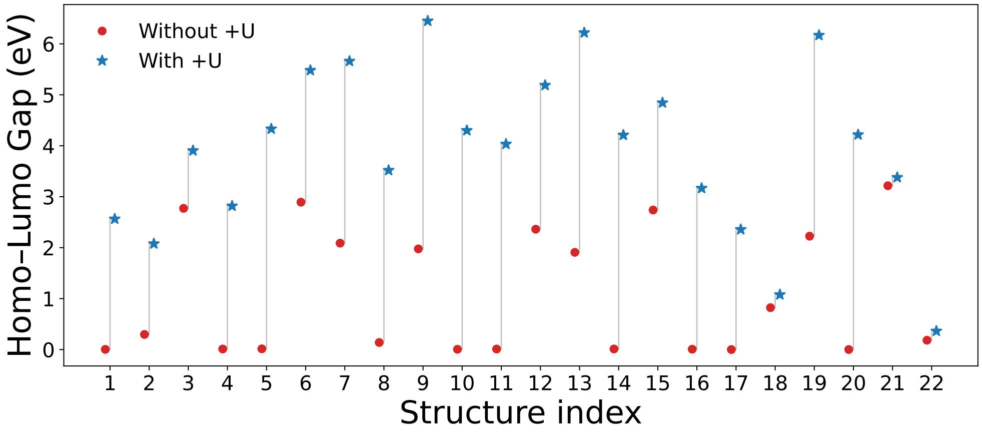

6. Assessment of Spin Contamination

Dataset from Pantazis et al. [1]

Formula	Multiplicity	Expected $\langle S^2 \rangle$	Calculated $\langle S^2 \rangle$
$[Fe(L)_2(OH)]$	6	8.7500	8.7568
$[Fe(L)_2(OH)]$	2	0.7500	0.7556
$[Fe(L)_2(NH_3)(OH)]$	6	8.7500	8.7539
$[Fe(L)_2(NH_3)(OH)]$	2	0.7500	0.7905
$[Fe(P)(OH)]$	6	8.7500	8.7540
$[Fe(P)(OH)]$	2	0.7500	0.7868
$[Fe(P)(OH)(NH_3)]$	6	8.7500	8.7549
$[Fe(P)(OH)(NH_3)]$	2	0.7500	0.7470
$[Fe(acac2trien)]^+$	6	8.7500	8.7550
$[Fe(acac2trien)]^+$	2	0.7500	0.7777
$[Fe(O)(NH_3)_5]^{2+}$	5	6.0000	6.0803
$[Fe(O)(NH_3)_5]^{2+}$	3	2.0000	2.0190
$[Fe(O)(NHC)]^{2+}$	5	6.0000	6.0844
$[Fe(O)(NHC)]^{2+}$	3	2.0000	2.0464
$[Fe(O)(NHC)(MeCN)]^{2+}$	5	6.0000	6.0840
$[Fe(O)(NHC)(MeCN)]^{2+}$	3	2.0000	2.0330
$[Fe(O)(TMC)(MeCN)]^{2+}$	5	6.0000	6.1062
$[Fe(O)(TMC)(MeCN)]^{2+}$	3	2.0000	2.0537
$[Fe(O_{ax})(PyTACN)(MeCN)]^{2+}$	5	6.0000	6.1019
$[Fe(O_{ax})(PyTACN)(MeCN)]^{2+}$	3	2.0000	2.0458
$[Fe(O_{eq})(PyTACN)(MeCN)]^{2+}$	5	6.0000	6.0959
$[Fe(O_{eq})(PyTACN)(MeCN)]^{2+}$	3	2.0000	2.0406
$[Fe(O)(N4Py)]^{2+}$	5	6.0000	6.0896
$[Fe(O)(N4Py)]^{2+}$	3	2.0000	2.0488

Dataset from Ruiz et al. [2]

Formula	Multiplicity	Expected $\langle S^2 \rangle$	Calculated $\langle S^2 \rangle$
$[Fe(3\text{-}OMe{-}salen)_2]^+$	2	0.7500	0.7732
$[Fe(3\text{-}OMe{-}salen)_2]^+$	6	8.7500	8.7545
$[Fe(acen)(NC_5H_3Me_2{-}3,4)2]$	5	6.0000	6.0894
$[Fe(acac)_2(trien)]^+$	2	0.7500	0.7775
$[Fe(acac)_2(trien)]^+$	6	8.7500	8.7551
$[Fe(phen)_2(NCS)_2]$	5	6.0000	6.0885
$[Fe(stpy)_4(NCS)_2]$	5	6.0000	6.0647
$[Fe(bpp)_3]^{2+}$	5	6.0000	6.0723
$[FeH2B(pz)2_2(bipy)]$	5	6.0000	6.0179
$[Fe(tzpy)_2(NCS)_2]$	5	6.0000	6.0442

Dataset from Truhlar et al. [3]

Formula	Multiplicity	Expected $\langle S^2 \rangle$	Calculated $\langle S^2 \rangle$
$[Fe(CO)_6]^{2+}$	5	6.0000	6.0544
$[Fe(CO)_6]^{2+}$	3	2.0000	2.0256
$[Fe(NCH)_6]^{2+}$	5	6.0000	6.0362
$[Fe(NCH)_6]^{2+}$	3	2.0000	2.0775
$[Fe(NH_3)_6]^{2+}$	5	6.0000	6.0005
$[Fe(NH_3)_6]^{2+}$	3	2.0000	2.0263
$[Fe(H_2O)_6]^{2+}$	5	6.0000	6.0003
$[Fe(H_2O)_6]^{2+}$	3	2.0000	2.0049
$[Fe(C_{10}H_8N_2)_3]^{2+}$	5	6.0000	6.0705
$[Fe(C_{10}H_8N_2)_3]^{2+}$	3	2.0000	2.0403
$Fe(C_6H_8N_2)_2Cl_2$	5	6.0000	6.0430
$Fe(C_6H_8N_2)_2Cl_2$	3	2.0000	2.0335
$[Fe(CO)_6]^{3+}$	6	8.7500	8.7507
$[Fe(CO)_6]^{3+}$	4	3.7500	3.7834
$[Fe(CO)_6]^{3+}$	2	0.7500	0.7973
$[Fe(CNH)_6]^{3+}$	6	8.7500	8.7515
$[Fe(CNH)_6]^{3+}$	4	3.7500	3.7753
$[Fe(CNH)_6]^{3+}$	2	0.7500	0.7946
$[Fe(NCH)_6]^{3+}$	6	8.7500	8.7511
$[Fe(NCH)_6]^{3+}$	4	3.7500	3.7892
$[Fe(NCH)_6]^{3+}$	2	0.7500	0.7825
$[Fe(NH_3)_6]^{3+}$	6	8.7500	8.7518
$[Fe(NH_3)_6]^{3+}$	4	3.7500	3.7848
$[Fe(NH_3)_6]^{3+}$	2	0.7500	0.7804
$[Fe(C_6H_{15}N_3)_2]^{3+}$	6	8.7500	8.7521
$[Fe(C_6H_{15}N_3)_2]^{3+}$	4	3.7500	3.7849
$[Fe(C_6H_{15}N_3)_2]^{3+}$	2	0.7500	0.7922

Dataset from Grimme et al.[4]

Formula	Multiplicity	Expected $\langle S^2 \rangle$	Calculated $\langle S^2 \rangle$	Calculated $\langle S^2 \rangle$ (+U)
$[Fe(CN)_6]^{3-}$	2	0.7500	0.7180	0.7907
$[Fe(CN)_6]^{3-}$	6	8.7500	8.7513	8.7542
$[Fe(CN)_6]^{4-}$	5	6.0000	6.0388	6.0025
$[FeF_6]^{3-}$	2	0.7500	0.7571	0.7572
$[FeF_6]^{3-}$	6	8.7500	8.7500	8.7500
$Fe(C_5H_5)_2$	5	6.0000	6.0167	6.0062
$[Fe(H_2O)_6]^{2+}$	5	6.0000	6.0004	6.0002
$[Fe(H_2O)_6]^{3+}$	2	0.7500	0.7584	0.7628
$[Fe(H_2O)_6]^{3+}$	6	8.7500	8.7504	8.7508
$[Fe(NH_3)_6]^{2+}$	5	6.0000	6.0005	6.0009
$[Fe(PH_3)_6]^{2+}$	5	6.0000	6.0382	6.0196
$[Fe(acac)_3]$	2	0.7500	0.7803	0.8070
$[Fe(acac)_3]$	6	8.7500	8.7612	8.7615
$[Fe(en)_3]^{2+}$	5	6.0000	6.0008	6.0010
$[Fe(C_5H_5)(CO)_3]^{+}$	3	2.0000	2.0141	2.1073

References

(1) M. Drosou, C. A. Mitsopoulou, and D. A. Pantazis, "Reconciling local coupled cluster with multireference approaches for transition metal spin-state energetics," *Journal of Chemical Theory and Computation*, **18**(6), 3538–3548, 2022.

(2) J. Cirera, M. Via-Nadal, and E. Ruiz, "Benchmarking density functional methods for calculation of state energies of first row spin-crossover molecules," *Inorganic Chemistry*, **57**(22), 14097–14105, 2018.

(3) P. Verma, Z. Varga, J. E. M. N. Klein, C. J. Cramer, L. Que, and D. G. Truhlar, "Assessment of electronic structure methods for the determination of the ground spin states of Fe (II), Fe (III) and Fe (IV) complexes," *Physical Chemistry Chemical Physics*, **19**(20), 13049–13069, 2017.

(4) H. Neugebauer, B. Bädorf, S. Ehlert, A. Hansen, and S. Grimme, "High-throughput

screening of spin states for transition metal complexes with spin-polarized extended tight-binding methods," *Journal of Computational Chemistry*, **44**(27), 2120–2129, 2023.
